# Integrating hand exoskeletons into goal-oriented clinic and home stroke and spinal cord injury rehabilitation

**DOI:** 10.1177/20556683221130970

**Published:** 2022-10-02

**Authors:** Aaron Yurkewich, Sara Ortega, José Sanchez, Rosalie H Wang, Etienne Burdet

**Affiliations:** 1 Bioengineering Department, Imperial College of Science Technology and Medicine, London UK; 2 Occupational Therapy, Centro Europeo de Neurociencias, Madrid, Spain; 3 Occupational Science and Occupational Therapy, 7938 University of Toronto, Toronto, ON, Canada

**Keywords:** robotic exoskeletons, upper extremity, assistive technology, home rehabilitation, stroke, spinal cord injury

## Abstract

**Introduction:**

Robotic exoskeletons are emerging as rehabilitation and assistive technologies that simultaneously restore function and enable independence for people with disabilities.

**Aim:**

We investigated the feasibility and orthotic and restorative effects of an exoskeleton-supported goal-directed rehabilitation program for people with hand impairments after stroke or Spinal Cord Injury (SCI).

**Method:**

A single-arm case-series feasibility study was conducted using a wearable untethered hand exoskeleton during goal-directed therapy programs with in-clinic and at-home components. Therapists trained stroke and SCI patients to use a hand exoskeleton during rehabilitation exercises, activities of daily living and patient-selected goals. Each patient received a 1-hour in-clinic training session on five consecutive days, then took the exoskeleton home for two consecutive days to perform therapist-recommended tasks. Goal Attainment Scaling (GAS) and the Box and Block Test (BBT) were administered at *baseline,* after *in-clinic* therapy and after *home* use, with and again without wearing the exoskeleton. The System Usability Scale (SUS), Motor Activity Log, and Fugl-Meyer Assessment were also administered to assess the intervention’s acceptability, adherence, usability and effectiveness.

**Results:**

Four stroke patients (Chedoke McMaster Stage of Hand 2–4) and one SCI patient (ASIA C8 Motor Stage 1) 23 ± 19 months post-injury wore the hand exoskeleton to perform 280 ± 23 exercise repetitions in the clinic and additional goal-oriented tasks at home. The patients performed their own goals and the dexterity task with higher performance following the 7-days therapy program in comparison to baseline for both exoskeleton-assisted (ΔGAS: 18 ± 10, ΔBBT: 1 ± 5) and unassisted (ΔGAS: 14 ± 14, ΔBBT: 3 ± 4) assessments. Therapists and patients provided ‘good’ SUS ratings of 78 ± 6 and no harmful events were reported.

**Conclusions:**

The exoskeleton-supported stroke and SCI therapy program with in-clinic and at-home training components was feasible.

## Introduction

People start their rehabilitation journeys with a diverse set of impairments, living environments, support networks, values and goals. The therapist provides personalized recommendations for rehabilitation exercises and assistive technology based on these considerations and the patient’s feedback and physical and cognitive state.^1–3^ Conventional rehabilitation techniques such as stretching and repetitive exercise are often used alongside modern approaches such as constraint induced movement therapy, neurodevelopmental therapy, bimanual training, mirror therapy and motor imagery to enhance function, independence, use of the affected limb, and attainment of patient-centred goals.^4–10^ However, it is difficult to keep patients with severe hand impairments (e.g., unable to open or close the hand) engaged in these therapies in clinic and home settings and motivated to use their affected hand in everyday situations after discharge home.^11,12^ This leads to non-use of the affected hand, increases in dystonia, spasticity and pain, declines in function, harmful compensatory motions, use of numerous one-handed assistive devices, and an inability to participate in leisure activities and activities of daily living.^13^ Service models that bridge the gap between assistive and rehabilitation robotics may enhance the intensity and duration of therapy and the care and support provided to people with hand impairments.

### Wearable robots in clinic and home rehabilitation programs

Robotic technologies are occasionally integrated into clinical practice to assist the affected upper extremity, which can be highly motivating to the patient, increase the number of exercise repetitions, and accelerate the recovery process.^9^ Few of these devices are integrated into the diverse set of clinical techniques that require patients to interact with real objects, as most of these robots assist the patient to steer toward an on-screen target.^14^ In addition, few of these devices are made accessible to patients in their everyday environment to provide ‘always-available’ assistance and goal-oriented rehabilitation.^15^

Wearable, untethered hand exoskeletons may provide a new avenue for rehabilitation and independent living, where the robot enhances the motion of those with severe impairments so they can participate in goal-directed exercises and activities in the clinic and at home that are typically reserved for less-affected patients. Assisting hand function may also encourage use of the hand and whole upper extremity, leading to broader recovery than only hand function as observed in previous hand rehabilitation studies.^16^ However, in practice there are numerous technical, clinical, patient-specific, and social barriers to creating these technologies and integrating them into clinical practice and home use. For instance, the device’s weight must be low and well-distributed as the gravitational forces will limit the weakened arm’s translational and rotational motions. Further, the device must provide strong forces to overcome muscle tone and tendon rigidity, while remaining comfortable on hands prone to joint instability and skin breakdown. Additionally, the device should be easy to put on and robust to control, while also giving the user sufficient support and flexibility to stabilize and manipulate various objects.^17^ Unique compromises are made in their design and control systems to ameliorate these usability barriers and enhance performance on unimanual and bimanual tasks, such as relocating the actuators and batteries to a backpack or waistbelt or reducing the controllable degrees of freedom.^18–25^ Few of these devices have reached the home feasibility trial stage, where the acceptability and adherence to use of the exoskeleton-assisted intervention could be measured and the facilitators to program completion could be identified.^26^ These devices have made further design compromises, such as eliminating active finger extension support or reducing the number of fingers supported.^27,28^ Further research is required to investigate:

• the *feasibility* (i.e. acceptability, adherence, usability, effectiveness) of integrating hand exoskeletons with five-finger extension and grip assistance into therapy programs with in-clinic and at-home components
• the *orthotic effect* (i.e. device-assisted performance) that hand exoskeletons provide during patient-specified goals and functional tasks after continued use
• the *restorative effect* (i.e. unassisted performance) after using hand exoskeletons during in-clinic and at-home components of rehabilitation programs that incorporate everyday objects

The purpose of this study is to assess the feasibility of integrating a custom-made robotic hand exoskeleton into clinic and home stroke and Spinal Cord Injury (SCI) rehabilitation programs and to appraise its orthotic and restorative potential before carrying out a larger clinical investigation. This research into the feasibility of using wearable robots in a myriad of in-clinic and at-home therapeutic techniques and exploring their orthotic and restorative potential is required to understand how to maximize the value added by wearable robots for motor recovery and goal attainment and identify areas to improve for future controlled clinical trials.

## Materials and methods

### Hand exoskeleton

**Figure 1.**
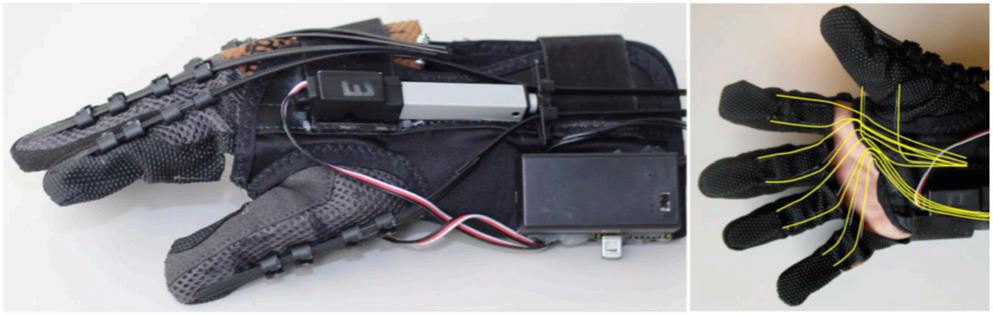
HERO is a battery-powered wearable untethered hand exoskeleton with (left) a dorsal actuator attached to five cables to provide five-finger extension and (right) a palmar actuator attached to 10 wires to provides five-finger grip assistance.

The Hand Extension Robot Orthosis Grip Glove (HERO) was iteratively designed with a team of engineers, therapists, and patients to provide hand extension assistance and then to additionally provide grip assistance.^22–24^ HERO is a wearable untethered hand exoskeleton that provides motion assistance for five-finger flexion, five-finger extension and thumb abduction, adduction and opposition. HERO’s battery, microcontroller, Inertial Measurement Unit (IMU), open-close control button, and actuators are mounted to a fabric wrist orthosis with an aluminum insert that supports the wrist in approximately 20^o^ of extension. The dorsal actuators are connected to flat cables on the dorsal side of the hand for extension and thumb abduction assistance. The ventral actuators are connected to flexible tendons on the palmar side for flexion and thumb opposition assistance that conforms to various object geometries. HERO has an open palm to ease donning. HERO’s hand opening and grasping assistance is triggered by either clicking the button with the opposite hand or moving the arm quickly (e.g., reaching, shaking, tapping) as detected by a pre-set IMU acceleration threshold. The therapist can train the patient to use either method as the second method requires arm function and is especially useful for bimanual tasks.^24^ Electromyography was used to control HERO in a previous study, however this was not feasible for a multi-day in-clinic and at-home therapy program as the control strategy required hands-on technical support, an external computer for communication, and greater setup and calibration time.^24^ HERO is shown in Figure 1, with further details on the open-source design, manufacturing process, and software provided in the supplementary material.

### Inclusion criteria

Sub-acute and chronic stroke patients with a Chedoke McMaster Stage of Hand^29^ assessment below 6 and SCI patients with an ASIA Upper Extremity Impairment Scale (ASIA-UE)^30^ below 5 for the finger flexors were recruited, such that their finger extension or grip strength was limited.

Patients were required to have an at-home caregiver if the therapist deemed this necessary for donning and using the device safely at home.

### Study design

A single-arm case-series feasibility study was conducted using HERO during goal-directed therapy programs with in-clinic and at-home components. Therapists at the Centro Europeo de Neurociencias rehabilitation clinic obtained ethics approval from their Center’s ethics review committee and completed the recruiting, assessing and training of the patients. Each patient provided informed consent to participate in the study. The bioengineers led the exoskeleton design, manufacturing and control aspects and instructed the therapists through video conferencing on how to use HERO, such as powering on and off, donning and doffing, controlling, sterilizing, monitoring for skin breakdown and adjusting the maximum ranges of motion assistance. Each patient received an additional 1-hour in-clinic therapy session with their therapist on five consecutive days, then took HERO home for two consecutive days. During the in-clinic sessions, the therapists were given the freedom to choose how to integrate HERO into practice, as in previous patient-centred therapy studies with splints and electrical stimulation,^31^ to train the patient to operate the device and assist them to perform rehabilitation exercises, activities of daily living and patient-selected goals more independently. Before bringing HERO home, the patient and therapist agreed on which Motor Activity Log-30 (MAL)^32^ tasks and patient-specific goals from their Goal Attainment Scaling (GAS)^33^ would be most relevant and safe to incorporate HERO into the home environment. The amount of prescribed home use was unconstrained, with the therapists prescribing several tasks that are expected to need 1 hour of use to complete, corresponding to the in-clinic dose. Tasks that would make the forearm wet were avoided since the electronics are not waterproof. Assessments were performed throughout the study to investigate the orthotic and restorative effects of exoskeleton-assisted therapy and the therapist and patient perspectives on the feasibility of using hand exoskeletons in therapy programs. The therapists used the Clinical Session Descriptions sheet, shown in the supplementary material, during each therapy session to manually record the types of tasks performed, the number of repetitions of each task, the incidents observed and the tasks recommended during home use.

### Assessments

At baseline, the GAS, Box and Block Test (BBT),^34^ Fugl-Meyer Upper Extremity Assessment (FM-UE),^35^ Functional Independence Measure (FIM)^36^ and either the Chedoke McMaster Stage of Hand and Stage of Arm (CM-H and CM-A) for stroke or ASIA-UE for SCI were administered without wearing HERO.

After 5 days of 1-hour in-clinic therapy with HERO, the *restorative effect* was assessed without wearing HERO using the GAS, BBT, FM-UE and FIM and the *orthotic effect* was assessed while wearing HERO using the GAS and BBT. *Feasibility* was assessed by the therapist and patient using separate System Usability Scale (SUS)^37^ and Quebec User Evaluation of Satisfaction with Assistive Technology Version 2.0 (QUEST)^38^ forms. They also completed descriptive questions on the negative and positive aspects of HERO and if HERO caused pain or skin discoloration. The Modified Ashworth Scale (MAS)^39^ was performed to assess the level of tone and spasticity in the index finger and identify if these factors affected HERO’s assistive capabilities. The MAL was assessed as a baseline for evaluating the patient’s adherence to the therapist’s home-use recommendations.

After 2 days of having access to HERO at home, the *restorative effect* was assessed without wearing HERO using the GAS and BBT, the *orthotic effect* was assessed while wearing HERO using the GAS and BBT, *adherence* was assessed using the MAL, and *feasibility* was assessed by the patient using the SUS and QUEST.

The restorative and orthotic effects compare the assessment scores after 5 days of in-clinic training and an additional 2 days of at-home training against the baseline scores. Statistical analyses are not conducted since this is a case-series feasibility study with a small sample size.

### Participants

Four sub-acute stroke patients and one SCI patient were recruited for this feasibility study. Their demographics at baseline are displayed in Table 1. The study included sub-acute and chronic patients with a range of hand and arm impairment severities. P3 was unable to raise the arm or move the fingers, while P1 was able to raise the arm and open and close the hand. There were three therapists in total as P1, P2, and P4 shared the same therapist.

**Table 1.** Demographics and baseline assessments for stroke and spinal cord injury patients.

Patient	Injury	Months post injury	CM-H	CM-A	FM-UE	*MAS	Affected/dominant hand	Gender	Age (years)
P1	Stroke	4	4	4	39	0	L/R	M	47
P2	Stroke	6	2	3	15	0	L/R	M	55
P3	Stroke	18	2	2	13	2	L/R	M	57
P4	SCI	44	n/a	n/a	50	0	R/R	F	27
P5	Stroke	41	2	3	33	1	R/L	F	51

ASIA-UE was used instead of CM for P4, with spinal level-scores of C2-5, C3-5, C4-5, C5-5, C6-4, C7-3, C8(finger flexors)-1(no active movement) and T1-1. *The MAS was performed after the 5th in-clinic session.

## Results

### Feasibility

#### Therapy delivery, acceptance and adherence

**Figure 2.**
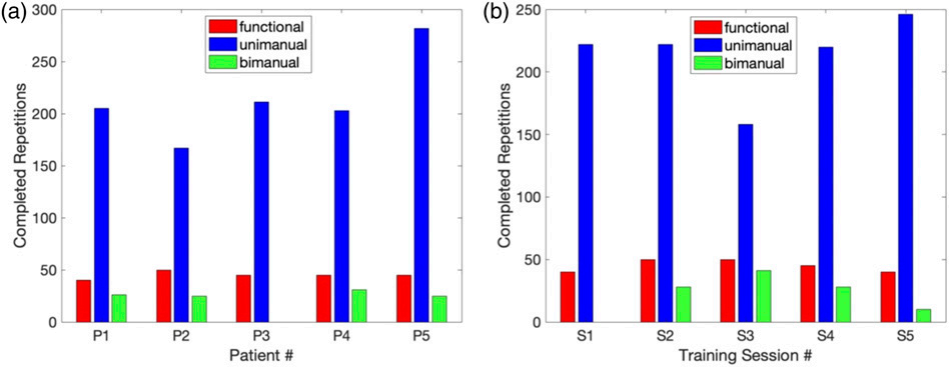
The five patients performed functional movements and unimanual and bimanual tasks during the five in-clinic exoskeleton-assisted therapy sessions, sub-divided by (a) patient and (b) session number.

Each patient completed all in-clinic training and at-home use components of the therapy program. During in-clinic sessions, the patients averaged 280 ± 23 repetitions while wearing HERO and completed additional repetitions without wearing HERO to observe changes in function. The tasks that the therapists chose to train can be categorized as functional movements (e.g., opening and closing the hand), unimanual (e.g., picking up and placing cones, dominoes, marbles, cubes, poker chips, clothes pegs and balls of various sizes, holding exercise weights, writing with a marker, grasping a cup and bringing it to the mouth and using a fork to pierce and eat grapes), and bimanual (e.g., opening a bottle, pouring water from a bottle into a cup and drinking from a cup, cutting food with a fork and a knife and bring it to the mouth, zipping up a jacket, peeling and eating a banana, making coffee and grasping and placing dishes on a table) tasks. The therapists started each session with HERO-assisted functional movements to warm-up the patient and get them accustomed to the assistance. On occasion, the therapists used readouts from an external electromyography system to synchronize the user’s intent to move with the triggering of exoskeleton assistance. The therapists mainly focused on unimanual tasks for the remainder of the session. After the first session, bimanual tasks were introduced into the training sessions for all patients except P3. Figure 2 shows the total number of functional movements and unimanual and bimanual tasks performed in the clinic across patients and sessions. The therapist reports on the tasks they administered in the clinic and prescribed for home practice are provided as supplementary material. An example video of how HERO was used in the clinic with P2 is also provided as supplementary material.

The therapists provided the patients, and their caregiver if needed, with instructions on how to put on, position, adjust, operate and take off HERO so that it could be used at home without therapist supervision. The therapists recommended using HERO for approximately 3 hours per day at home to perform everyday tasks, which included 10–16 tasks from the MAL per participant (e.g., open a drawer, hold a phone, pick up a cup, use a fork, put on socks, write) and additionally the participants’ specific goals (e.g., opening a contact lens bottle, grasping a glass or bottle and bringing it to their mouth, lowering their trousers, cutting food with a fork and knife, zipping up a sweatshirt, using a computer keyboard, using kitchen appliances, setting the table, removing clothes from the drawer, brushing teeth with a toothbrush).

#### Usability

The patients rated HERO as having ‘good’ usability, according to the SUS, after the in-clinic component (SUS 70% ± 8) and provided higher ratings after using HERO at home (SUS 78% ± 7). All patients except P3 learned to put on and use HERO independently. P3 had considerable hand tone and spasticity and arm weakness, so P3’s caregiver was instructed to help in putting on HERO and supporting the arm. After home use, P3 provided the highest rating of 90% while P5 provided the lowest rating of 72%. The patients’ therapists similarly rated HERO as having ‘good’ usability (78% ± 7). The therapists provided the highest rating of 85% for P2 and the lowest rating of 68% for P1.

The patients provided an average QUEST rating between ‘quite satisfied’ and ‘more or less satisfied’ after the in-clinic (QUEST 3.5 ± 0.5) and at-home (3.4 ± 0.7) components, and the therapists rated similarly (3.3 ± 0.3). After home use, P3 provided the highest rating of 4.2 and P2 provided the lowest rating of 2.3. Consistent with the SUS, the therapists provided the highest rating for P2 of 3.5 and the lowest rating for P1 of 2.8. The patients were ‘quite satisfied’ with the ease of use of HERO, the therapy delivered using HERO, and the quality of professional services received. The patients and therapists were satisfied with the device’s comfort and safety as their ratings remained above 70% on the QUEST for these categories. They were ‘not very satisfied’ with the ease in adjusting HERO (i.e. putting on, taking off, fixing, fastening). The SUS and QUEST results are provided in Table 2.

**Table 2.** Patients’ and therapists’ usability ratings for the HERO-assisted therapy program.

Patient	SUS			QUEST		
	P-C	P-H	T-C	P-C	P-H	T-C
P1	72.5	75.0	67.5	3.1	3.2	2.8
P2	75.0	75.0	85.0	3.8	2.3	3.5
P3	72.5	90.0	75.0	4.1	4.3	3.4
P4	55.0	80.0	82.5	2.9	3.3	3.3
P5	72.5	72.5	80.0	3.8	3.7	3.3
MEAN	69.5	78.5	78.0	3.5	3.4	3.3

Assessments were performed by the patient after the in-clinic component (P-C), the patient after the at-home component (P-C) and the therapist after the in-clinic component (T-C).

There were no reports of safety issues (e.g., pain, discomfort, harmful task errors) or adverse events, pain or skin breakdown, which the therapist checked after each in-clinic session and at the follow-up assessment session after home use. The patients and therapists responded that HERO did not cause any pain at any time points. HERO left small marks on some patients’ skin after use and these marks disappeared within 10 min. All the negative and positive aspects reported by patients or therapists for HERO are paraphrased below, with similar responses given by both groups.

Negative aspects reported for HERO were:

• (donning) difficulty putting the device on clenched hands and adjusting cable tensions
• (assistance) incomplete and inconsistent finger extension across fingers, mechanical components interfering with sensation, hand movement, and grasping large objects, insufficient grip strength for some tasks, inability to assist individual fingers or adjust the speed or amount of motion, reduction in arm motion for weak arms due to device weight
• (control) the arm acceleration control mode did not always trigger, the button was difficult to reach, the assistance was not synchronized with the user’s intent to move
• (inconveniences) short battery life, ill-fitting thumb, poor appearance, not waterproof

Positive aspects reported for HERO were:

• (independence) ability to consistently and completely open and close the hand, grasp small items, grasp heavier objects, move objects with precision, perform functional activities, perform daily activities with less difficulty, perform daily activities that could not be completed without HERO (especially with small items)
• (therapy) allows the user to carry out movements when they do not have enough capacity to open and close the hand, engage and practice with the affected upper limb, increase the number of repetitions, use the hand and arm together, work on bimanual activities and tasks that require fine manipulative dexterity, and integrate the hand into activities of daily life
• (versatility) ability to be used with external electromyography and electrical stimulation systems, ability for two HEROs to be used at once to support both hands
• (accessibility) easy to use, portable, convenient, inexpensive, and lightweight

#### Orthotic effect

All patients performed their own goals with higher performance following the in-clinic (ΔGAS: 22 ± 2) and at-home (ΔGAS: 18 ± 10) components of the therapy program while wearing HERO in comparison to at baseline without wearing HERO. P4 showed the largest improvement, scoring 37 at baseline and 71 after the program. P2, P3 and P5 scored higher after the in-clinic component than after the additional at-home component. The difference in GAS score was also calculated between wearing and not wearing HERO after the in-clinic component (ΔGAS In-Clinic: 16 ± 9) and in-home component (ΔGAS At-Home: 3 ± 15) to remove impairment restoration effects. After the program, P2, P4, and P5 performed better on the GAS while wearing HERO and P1 and P3 performed better without wearing HERO.

The patients without hand extension (P2, P3, P5) were unable to transfer any blocks during the BBT without HERO. With HERO, they transferred an average of three blocks after in-clinic training and five blocks after the additional in-home training. P1 and P4 were able to open and close their hand and transferred more blocks without HERO than with HERO.

Each patient showed enhanced performance on the MAL-Amount of Use (MAL-AOU) and MAL-How Well (MAL-HW) assessments with HERO compared to without HERO (ΔMAL-AOU: 0.5 ± 0.6, ΔMAL-HW: 0.5 ± 0.4). P5 showed the largest improvement on the MAL-AOU and MAL-HW, scoring 0.8 and 0.8 without HERO and 2.3 and 2.0 with HERO. The FIM assessment was discontinued because HERO can only be used for four of the six tasks since it is not waterproof and since the FIM tasks require a broad range of trunk and upper and lower limb capabilities that HERO alone would not remediate. The orthotic effects from the GAS, BBT and MAL assessments are shown in Table 3.

**Table 3.** Orthotic effects of HERO during the in-clinic and at-home therapy program.

Patient	GAS			BBT			MAL-AOU		MAL-HW	
	B	C-H	H-H	B	C-H	H-H	C	H-H	C	H-H
P1	31.4	50.0	50.0	11	5	5	1.7	1.8	1.6	1.8
P2	22.6	45.4	31.7	0	3	5	1.5	2.0	0.8	1.1
P3	37.7	62.3	47.5	0	3*	3*	0.8	0.9	0.9	1.2
P4	37.2	60.3	70.5	14	8	11	3.0	3.6	2.9	3.6
P5	36.3	59.1	54.6	0	4	6	0.8	2.3	0.8	2.0
MEAN	33.0	55.4	50.9	5	5	6	1.6	2.1	1.4	1.9

Assessments were performed at baseline without wearing HERO (B), after the in-clinic component without wearing HERO (C), after the in-clinic component while wearing HERO (C-H) and after the at-home component while wearing HERO (H-H). *Assessment was performed with the arm supported by a therapist.

### Restoration effect

**Figure 3.**
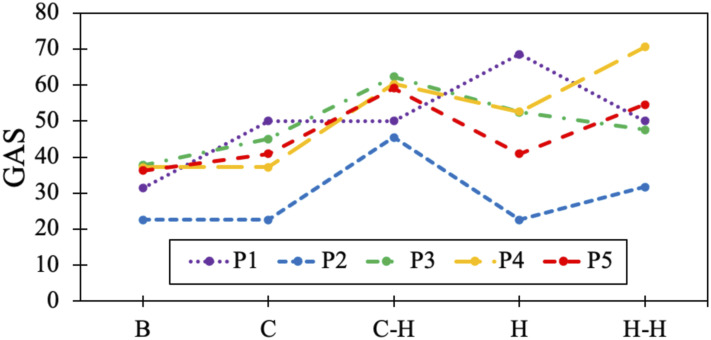
The GAS was performed at baseline without wearing HERO (B), after the in-clinic component without wearing HERO (C), after the in-clinic component while wearing HERO (C-H), after the at-home component without wearing HERO (H) and after the at-home component while wearing HERO (H-H).

Without wearing HERO, the patients on average performed their own goals with higher performance following the in-clinic (ΔGAS: 6 ± 8) and at-home (ΔGAS: 14 ± 14) components in comparison to at baseline. P1 was in the sub-acute stage post-stroke and showed the largest improvement from 31 to 69 points. P3, P4 and P5 improved to a lesser extent and P2 showed no improvement. On average, the participants performed the functional block-transfer task with higher performance following the in-clinic (ΔBBT: 1.4 ± 2.2) and at-home (ΔBBT: 3 ± 4.1) components. P1 and P4 improved from 11 and 14 blocks to 19 and 21 blocks, while P2, P3 and P5 did not transfer any blocks. The participants showed minimal change on the FM-UE (ΔFM-UE 0.6 ± 2.7), with P1 showing the largest improvement of 4 points. On average, there was a small decline in function on the shoulder and elbow section (ΔFM-SE -1.0 ± 2.1) and a small increase on the wrist (ΔFM-W 0.4 ± 0.5), hand (ΔFM-H 0.6 ± 0.9) and coordination (ΔFM-C 0.6 ± 1.3) sections. The restorative effects from the GAS, BBT and FM assessments are shown in Table 4. All GAS results are plotted in Figure 3.

**Table 4.** Restoration effects of the HERO-assisted in-clinic and at-home therapy program.

Patient	GAS			BBT			FM-UE		FM-SE		FM-WH	
	B	C	H	B	C	H	B	C	B	C	B	C
P1	31.4	50.0	68.6	11	16	19	39	43	22	24	14	16
P2	22.6	22.6	22.6	0	0	0	15	14	12	11	0	0
P3	37.7	45.1	52.5	0	0	0	13	15	11	8	2	4
P4	37.2	37.2	52.6	14	16	21	50	51	33	33	11	12
P5	36.3	40.9	40.9	0	0	0	33	30	24	21	7	7
MEAN	33.0	39.2	47.4	5	6	8	30.0	30.6	20.4	19.4	6.8	7.8

Assessments were performed without wearing HERO at baseline (B), after the in-clinic component (C) and after the at-home component (H).

## Discussion

Integrating the HERO hand exoskeleton into the in-clinic and at-home components of a therapy program was feasible for stroke and SCI patients in this study. Importantly, there were no safety issues reported with the device’s use in clinic or home settings with these patients. Each patient performed personalized meaningful goals (GAS) and a functional task (BBT) with greater success following the therapy program compared to their baseline, with three patients performing best while wearing HERO and two patients performing best without wearing HERO. Key contributions of this study were that:

• All patients completed the in-clinic and at-home components of the hand exoskeleton-assisted therapy program without experiencing any painful or unsafe events
• Providing the exoskeleton for home use along with a set of task recommendations was effective in increasing the amount of use and quality of motion of the affected upper extremity for all patients
• The hand exoskeleton may be accepted into clinical practice given its promising usability ratings from the patients and therapists
• Areas of improvement have been identified to facilitate greater adoption of the exoskeleton into clinic and home therapy programs

In addition, three of five patients demonstrated enhanced performance on goal-oriented activities and the functional task with hand exoskeleton assistance (*orthotic effect*) and four of five patients demonstrated enhanced performance on goal-oriented activities following the program without hand exoskeleton assistance (*restorative effect*). These results are supported by a small retrospective study that also found that using an upper-extremity exoskeleton in clinic and home therapy is safe and can restore motor function^40^; however, these orthotic and restorative effects should be interpreted with care as larger sample sizes are required to validate these findings.

### Focusing on goal attainment

Goal Attainment Scaling is commonly used in occupational therapy to measure the effectiveness of a therapeutic intervention.^41^ Occupational therapists are trained to interview and physically assess the patient to define goals that are personalized to the patient’s needs and attainable to some extent within the therapeutic period. These goals are used to engage the patient and orient the therapist’s decision making toward restorative and compensatory strategies and assistive technology procurements that provide the greatest benefit to the patient. However, exoskeleton therapy studies rarely report GAS results and often specify rigid and impersonalized therapy protocols that are difficult to integrate into practice. This results in a divide between clinical practice and exoskeleton-assisted therapy research.

An occupation-based therapy model was utilized for this study, in which the GAS was chosen as the main outcome measure to enable therapists to use their clinical judgement to select in-clinic and at-home rehabilitation tasks that help each patient attain their goals.^42^ The therapists used their prior experience and readily available objects and rehabilitation technologies to develop diverse and personalized therapy tasks (i.e., 38 different in-clinic tasks and 29 different home tasks were prescribed), which would have been difficult to encompass in a virtual game or one-size-fits-all therapy protocol. This approach led to a delivery of therapy that patients were ‘very satisfied’ with and that was transferable to patients with multiple injury types and severity levels. Using person-centred assessment metrics already performed as part of standard practice could also enable more cost-effective and timely clinical trials and greater interpretability of the results.^43,44^ Further, including patient reported outcomes as tools in exoskeleton-assisted therapy puts the focus on the patient’s needs and motivates the combination of impairment restoration techniques, safe compensatory strategies and assistive technologies to maximize independence and provide personalized care.

### Impact of wearable exoskeletons on therapy delivery

The hand exoskeleton’s assistance enhanced the patients’ ability to perform their personalized goals while they were unable to open or close their hand. The exoskeleton’s wearability allowed for tasks to be completed in various postures and environments. These aspects enabled the patients to practice goal-directed upper-extremity tasks and use the upper-extremity more regularly at home, for tasks such as grasping and drinking from a cup, eating lunch with a fork and knife, and writing. This gave the therapists more engaging options for how to train the affected upper extremity, as otherwise tasks would need to be modified extensively or replaced with a range of motion exercises. The therapists were satisfied with the technology-enhanced therapy program as they were able to assign a variety of reach and grasp tasks and bimanual tasks that aligned with their patients’ interests, motivating the use of therapist-directed and goal-oriented therapy programs in clinical studies with exoskeletons.

### Feasibility of exoskeletons during therapy programs

This study showed that goal-oriented therapy programs that integrate exoskeletons are feasible for those with moderate or severe hand impairment. The usability results from this study were similar to previous studies with this device,^23,24^ suggesting that using the device at home did not negatively affect the usability results. These usability results are supported by a small home pilot study that found that three of four stroke participants were satisfied with a different hand exoskeleton.^45^ A list of hand exoskeletons that have been evaluated with stroke and SCI patients is provided as a supplementary table. The usability ratings were on average higher with HERO, which may be due to the additional training provided and enhanced portability and orthotic effects observed. Interestingly, there were individual differences between the patient and therapist usability ratings, while their average usability ratings were similar. The SCI patient’s usability ratings were within the range provided by the stroke patients’, suggesting that hand exoskeletons are useable across neurological populations; however, usability studies with larger sample sizes are required for both populations. Future studies should also include follow-up interviews to detail the participants’ perceptions of HERO’s usability, such as why the two stroke patients that performed better on the GAS without wearing HERO provided positive SUS scores and why most participants provided higher usability ratings after taking the device home. The users identified donning, assistance, and control inconveniences with the design, which were overcome through therapist and caregiver support; however, these considerations should be addressed in future exoskeleton designs to reach ‘excellent’ levels of usability (i.e., above 85 on the SUS). These findings should guide engineers and clinicians to design wearable robots and therapy programs with enhanced usability and motivate future studies with larger sample sizes, longer therapeutic durations, and semi-structured interviews to generalize findings related to severity, injury type and usability and capture technology adoption barriers related to environmental and social factors.

### Device limitations

Device usability barriers were overcome to some extent by including an in-clinic training component; however, design improvements should be made to address critiques provided by the therapists and participants, such as making the device easier to put on, relocating materials that block fingertip and palm sensation, increasing grip strength, and controlling the device safely through user intent. Motion, force and electromyography sensors and data recording capabilities could benefit the exoskeleton’s usability by enabling the exoskeleton assistance to be controlled by the user’s volition and allow on-line assessments to be performed that supplement intermittent clinical assessments and provide feedback on user adherence and safety to the therapist. The hand exoskeleton could then be integrated with additional upper and lower extremity exoskeletons, as well as electrical stimulation and augmented reality games, to enhance assistance and sensory feedback and investigate the usability and effectiveness of rehabilitation technology hybrids.

### Considerations for future clinical studies

Four patients attained their goals to a greater degree following the program when assessed without wearing the hand exoskeleton and three patients showed improvement on the FM-WH. These improvements may result from movement restoration and strengthening; however, test-retest variability and changes in impairment presentation between days could also contribute to these promising results. The FM-UE was chosen for this protocol as it has distinct sections for measuring shoulder-elbow or wrist or hand function, though additional assessments such as the Action Research Arm Test, Wolf Motor Function Test and Chedoke Arm and Hand Activity Inventory could be added to assess performance on standardized object manipulation tasks. Further assessments of motivation and cognitive ability could be added to understand how these aspects affect device usability, program adherence and motor recovery. These feasibility study results are useful for determining the effect size for future studies; however, the sample size of this study is too small to provide statistical results of its effectiveness directly. Future studies are needed to assess the effectiveness of the therapy program in comparison to standard care and other technology-enhanced programs, which will require control groups (i.e. groups that are not given access to the device), larger sample sizes and separate acute, sub-acute or chronic groups to assess effects statistically. The 1-week study duration is most likely too short to show changes in motor recovery and should be lengthened as therapy programs generally require 8–12 weeks and 6 hours of therapy per day to produce significant and clinically meaningful changes in impairment restoration^5,31^ and the assessors of recovery should be blinded to remove bias. The greatest usability ratings and assistive benefits were realized by patients with residual arm function that were unable to open or close the hand in both stroke or SCI populations, making this a key study population for understanding the benefits of wearable untethered hand exoskeletons.

## Conclusions

The hand exoskeleton enabled people with hand impairments to perform a variety of functional movements, unimanual and bimanual tasks and goal-directed activities more independently. Integrating exoskeletons, such as HERO, into in-clinic and at-home rehabilitation programs is feasible and may enable greater independence in everyday activities and greater access to rehabilitation. This is a feasibility study with a small sample size that sets the stage for clinical trials that integrate exoskeletons more broadly into rehabilitation and everyday activities to understand their effects on patient engagement, motor recovery, independence, and quality of life.

## Supplemental Material

Supplemental Material - Integrating hand exoskeletons into goal-oriented clinic and home stroke and spinal cord injury rehabilitation Click here for additional data file. Supplemental Material for Integrating hand exoskeletons into goal-oriented clinic and home stroke and spinal cord injury rehabilitation by Aaron Yurkewich, Sara Ortega, José Sanchez, Rosalie H Wang and Etienne Burdet in Journal of Rehabilitation and Assistive Technologies Engineering

**Figure:** Video 1
