# Supplementary material for: Integrating hand exoskeletons into goal-oriented clinic and home stroke and spinal cord injury rehabilitation
Source: J Rehabil Assist Technol Eng. 2022 Oct 2;9:20556683221130970. doi: 10.1177/20556683221130970 (PMC9535266; doi:10.1177/20556683221130970)
Supplement: Supplemental Material - Integrating hand exoskeletons into goal-oriented clinic and home stroke and spinal cord injury rehabilitation [file sj-pdf-1-jrt-10.1177_20556683221130970.pdf]

SUPPLEMENTARY TABLE I. WEARABLE HAND ROBOT USE CASES EVALUATED WITH STROKE AND SPINAL CORD INJURY (SCI) PATIENTS

Device	Actuation (#, Type)	Weight: Hand, Total	Grasp Force	Finger Extension Force	Fingers Actuated (#)	Wrist Supp-orted	Tethering	Population Evaluated (#, use case)	Therapy Tasks	Control Signal Used	Don Time (min)	Orthotic Effects, relative to baseline	Restorative Effects, relative to baseline
**HERO Glove (this article)**	**2, motors, screw-drive, cables**	**284g**	**12.7N**	**26.7N**	**5**	**Yes**	**Fully Portable**	**4 Stroke, 1 SCI, 5 in-clinic and 2 at-home therapy sessions**	**Patient-specific goals using real objects**	**Button, IMU**	**3**	**GAS +17.9, BBT +1, MAL-AOU +0.5, MAL-QOM + 0.5, SUS: 72-90**	**GAS +14.4, BBT +3, FM-UE +0.6**
Hand-In-Motion [1]	5, motors, cables	500g, 3000g	n/a	n/a	3	n/a	Waist	5 Stroke, 6 weeks of in-clinic therapy or home use	Virtual tasks or Patient selected tasks with real objects	Motion, Force	n/a	Jebsen-Taylor Hand Function Test, no benefit, SUS: 38-100	Grip strength -3 to +15%
MyoPro Motion-G [2][3]	2, motors, links, elbow, wrist & hand	n/a, 1800g	n/a	n/a	2	No	Fully Portable	18 Stroke, 1 session; 9 Stroke, 12 weeks of group and home therapy	Group therapy and home practice using real objects	EMG, Buttons	n/a	FM-UE +8.72, 13%, Able to grasp cup, fork and laundry basket	FM-UE +7.3, MAS – 0.6
X-Glove [4][5]	5, motors, screw-drive, cables	375g, 800g	0N (est.)	60N	4	No	Back	13 & 22 Stroke, 15 in-clinic therapy sessions	Standardized exercises with real objects	Force, EMG, Voice	10		FM-UE 0; Graded-WMFT -10.1s, ARAT +2.6; CAHAI +0.67
Hand of Hope [6][7]	5, motors, screw-drive, links	459g, n/a	12N (pinch)	23N (est.)	5	Yes	Table, Outlet	10, stroke therapy, 20 sessions	Virtual tasks	EMG	n/a		FM-UE +5.2, ARAT +14.4, WMFT +9.4
Cheng *et al.* [8]	8, solenoids, pneumatic tubes	n/a	n/a	n/a	4	No	Table, Outlet	5 Stroke, 18 2hr in-clinic therapy sessions	Virtual tasks	BCI, Button	n/a		FMA-UE +4.8, ARAT 2.2
Gloreha Lite [9]	5, motors, cables	80g, 5000g	5N (est. [1])	12N (est.)	5	Yes	Trolley, Outlet	21 Sub-acute Stroke, ~40 in-clinic therapy sessions	Virtual tasks	Game-Directed	n/a		Motricity Index +12; Peg Test
Gasser *et al.* [10]	2, motors, cables, links	400g	50N (grasp)	n/a	4	Yes	Fully Portable	1 Stroke, 1 session		Button	n/a	Grasped water bottle faster	
Butzer *et al.* [11]	4, motors, screw-drive, cables	113g, 867g	6.4N per finger	6.4N per finger	4	Yes	Back	1 Stroke, 1 SCI, 1 session		Buttons	2	ARAT -1 and +15, SUS 30-65	
Exo-Glove [12], [13]	1, motor, cables, clutch	194g, n/a	40N (power)	16.7N (est.)	3	Yes	Table	1 SCI, 1 session		Button, Motion, Vision	n/a	Able to grasp 8 objects (Not compared to unassisted)	
Maestro [14], [15]	8, motors, cables, links	205g, n/a	n/a	n/a	3	Yes	Table, Outlet	2 SCI, 1 session		EMG	n/a	Sollerman Test +5	
Soft Robotic Glove [16]–[18]	7, solenoids, pneumatic tubes	77g, 5000g	15N (grasp)	n/a	5	Yes	Table, Outlet	1 Muscular Dystrophy, 9 SCI, 1 session		Buttons, EMG	n/a	TRI-Hand Function Test, 34%	
Soekadar *et al.* [19]	4, motors, cables, links	438g, n/a	4N (pinch)	5N (est.)	2	Yes	Wheel-chair	6 SCI, 1 session		EEG + EOG	5 (est.)	TRI-Hand Function Test, +18%	
BiomHED [20]	7, motors, cables	1000g, n/a	n/a	2.5N (est.)	5	No	Table, Outlet	4 Stroke, 1 session		n/a	5-10	Improved index finger workspace and kinematics	
Park *et al.* [21]	2, motors, screw-drive, cables	n/a, n/a	n/a	**10N**	4	No	Table, Outlet	5 Stroke, 1 session		Button	n/a	Able to grasp water bottle	
Yap *et al.* [22]	5, solenoids, pneumatic tubes	180g, 1260g	8.4N (pinch)	4.5N	5	Yes	Table	2 Stroke, 1 session		Button	n/a	Water bottle and can grasped faster	
SPAR Glove. [23]	7, motors, cables	220g, 16000g	83N	n/a	5	No	Table, Outlet	2 SCI, 1 session		EMG	n/a	Able to detect multiple grasp poses	

REFERENCES

[1] Prange-Lasonder GB, Radder B, Kottink AI, Melendez-Calderon A, Buurke JH, Rietman JS. Applying a soft-robotic glove as assistive device and training tool with games to support hand function after stroke: Preliminary results on feasibility and potential clinical impact. In2017 International Conference on Rehabilitation Robotics (ICORR) 2017 Jul 17 (pp. 1401-1406). IEEE.

[2] Peters HT, Page SJ, Persch A. Giving them a hand: wearing a myoelectric elbow-wrist-hand orthosis reduces upper extremity impairment in chronic stroke. Archives of physical medicine and rehabilitation. 2017 Sep 1;98(9):1821-7.

[3] McCabe JP, Henniger D, Perkins J, Skelly M, Tatsuoka C, Pundik S. Feasibility and clinical experience of implementing a myoelectric upper limb orthosis in the rehabilitation of chronic stroke patients: a clinical case series report. PloS one. 2019 Apr 12;14(4):e0215311.

[4] Fischer HC, Triandafilou KM, Thielbar KO, Ochoa JM, Lazzaro ED, Pacholski KA, Kamper DG. Use of a portable assistive glove to facilitate rehabilitation in stroke survivors with severe hand impairment. IEEE Transactions on Neural Systems and Rehabilitation Engineering. 2015 Dec 31;24(3):344-51.

[5] Thielbar KO, Triandafilou KM, Fischer HC, O'Toole JM, Corrigan ML, Ochoa JM, Stoykov ME, Kamper DG. Benefits of using a voice and EMG-driven actuated glove to support occupational therapy for stroke survivors. IEEE Transactions on Neural Systems and Rehabilitation Engineering. 2016 May 17;25(3):297-305.

[6] Susanto EA, Tong RK, Ockenfeld C, Ho NS. Efficacy of robot-assisted fingers training in chronic stroke survivors: a pilot randomized-controlled trial. Journal of neuroengineering and rehabilitation. 2015 Dec;12(1):1-9.

[7] Hu XL, Tong KY, Wei XJ, Rong W, Susanto EA, Ho SK. Coordinated upper limb training assisted with an electromyography (EMG)-driven hand robot after stroke. In2013 35th Annual International Conference of the IEEE Engineering in Medicine and Biology Society (EMBC) 2013 Jul 3 (pp. 5903-5906). IEEE.

[8] Cheng N, Phua KS, Lai HS, Tam PK, Tang KY, Cheng KK, Yeow RC, Ang KK, Guan C, Lim JH. Brain-computer interface-based soft robotic glove rehabilitation for stroke. IEEE Transactions on Biomedical Engineering. 2020 Apr 2;67(12):3339-51.

[9] Bernocchi P, Mulè C, Vanoglio F, Taveggia G, Luisa A, Scalvini S. Home-based hand rehabilitation with a robotic glove in hemiplegic patients after stroke: a pilot feasibility study. Topics in stroke rehabilitation. 2018 Feb 17;25(2):114-9.

[10] Gasser BW, Bennett DA, Durrough CM, Goldfarb M. Design and preliminary assessment of vanderbilt hand exoskeleton. In2017 International Conference on Rehabilitation Robotics (ICORR) 2017 Jul 17 (pp. 1537-1542). IEEE.

[11] Bützer T, Lambercy O, Arata J, Gassert R. Fully wearable actuated soft exoskeleton for grasping assistance in everyday activities. Soft Robotics. 2021 Apr 1;8(2):128-43. [12] H. In and K. Cho, "Exo-Glove : Soft wearable robot for the hand using soft tendon routing system," IEEE Robot. Autom., vol. 22, no. March 2015, pp. 97–105, 2015.

[13] Kim D, Kang BB, Kim KB, Choi H, Ha J, Cho KJ, Jo S. Eyes are faster than hands: A soft wearable robot learns user intention from the egocentric view. Science Robotics. 2019 Jan 30;4(26):eaav2949.

[14] Yun Y, Dancausse S, Esmatloo P, Serrato A, Merring CA, Deshpande AD. An EMG-driven assistive hand exoskeleton for spinal cord injury patients: Maestro. InIEEE International Conference on Robotics and Automation (ICRA) 2017.

[15] Yun Y, Agarwal P, Fox J, Madden KE, Deshpande AD. Accurate torque control of finger joints with UT hand exoskeleton through Bowden cable SEA. In2016 IEEE/RSJ International Conference on Intelligent Robots and Systems (IROS) 2016 Oct 9 (pp. 390-397). IEEE.

[16] Cappello L, Meyer JT, Galloway KC, Peisner JD, Granberry R, Wagner DA, Engelhardt S, Paganoni S, Walsh CJ. Assisting hand function after spinal cord injury with a fabric-based soft robotic glove. Journal of neuroengineering and rehabilitation. 2018 Dec;15(1):1-0.

[17] Polygerinos P, Galloway KC, Sanan S, Herman M, Walsh CJ. EMG controlled soft robotic glove for assistance during activities of daily living. In2015 IEEE international conference on rehabilitation robotics (ICORR) 2015 Aug 11 (pp. 55-60). IEEE.

[18] Polygerinos P, Wang Z, Galloway KC, Wood RJ, Walsh CJ. Soft robotic glove for combined assistance and at-home rehabilitation. Robotics and Autonomous Systems. 2015 Nov 1;73:135-43.

[19] Soekadar SR, Witkowski M, Gómez C, Opisso E, Medina J, Cortese M, Cempini M, Carrozza MC, Cohen LG, Birbaumer N, Vitiello N. Hybrid EEG/EOG-based brain/neural hand exoskeleton restores fully independent daily living activities after quadriplegia. Science Robotics. 2016 Dec 6;1(1):eaag3296.

[20] Lee SW, Landers KA, Park HS. Development of a biomimetic hand exotendon device (BiomHED) for restoration of functional hand movement post-stroke. IEEE Transactions on Neural Systems and Rehabilitation Engineering. 2014 Jan 13;22(4):886-98.

[21] Park S, Bishop L, Post T, Xiao Y, Stein J, Ciocarlie M. On the feasibility of wearable exotendon networks for whole-hand movement patterns in stroke patients. In2016 IEEE international conference on robotics and automation (ICRA) 2016 May 16 (pp. 3729-3735). IEEE.

[22] Yap HK, Lim JH, Nasrallah F, Yeow CH. Design and preliminary feasibility study of a soft robotic glove for hand function assistance in stroke survivors. Frontiers in neuroscience. 2017 Oct 9;11:547.

[23] Britt JE, O'Malley MK, Rose CG. Electromyographic Classification to Control the SPAR Glove. IFAC-PapersOnLine. 2021 Jan 1;54(20):244-50.
